# Gold-Induced Chemical Perturbations in CdTe-Based Photovoltaic Cells

**DOI:** 10.1021/acsami.5c24315

**Published:** 2026-03-20

**Authors:** Ryan Muzzio, Darius Kuciauskas, B. Edward Sartor, Joshua A. Brown, Jalal Nawash, Joel N. Duenow, Hongling Lott, Chungho Lee, Matthew O. Reese, Craig L. Perkins

**Affiliations:** † National Laboratory of the Rockies (NLR), Golden, Colorado 80401, United States; ‡ School of Mechanical and Materials Engineering, 6760 Washington State University, Pullman, Washington 99164, United States; § California Technology Center (CTC), 338440 First Solar Inc, Santa Clara, California 95050, United States

**Keywords:** photovoltaics, native oxide, metallization, photoemission, auger, photoluminescence, CdTe

## Abstract

Back-contacting p-type CdTe has been identified as one of the major areas of loss in CdTe photovoltaic (PV) power conversion efficiency (PCE). In research settings, Au is a common contact material due to its ease of use and decent performance. This work provides a detailed investigation into using gold for back contacting As-doped, CdCl_2_-treated, polycrystalline CdTe that has been exposed to air after absorber processing, another routine practice. First, X-ray photoemission spectroscopy (XPS) is used to determine the native oxide to be 1.6 nm of CdTeO_3_ by using a combination of angle-resolved XPS and the cadmium-modified Auger parameter. During gold metallization of CdTe, oxygen and oxidized tellurium are eliminated from the thin CdTeO_3_ native oxide. The fates of the released oxygen and possibly cadmium and tellurium are not known, but these reaction byproducts can enter the absorber bulk or grain boundaries, stay at the interface, or dissolve in the Au. Interfacial hole barriers between CdTe and Au are measured for samples with and without the native oxide present prior to metallization. Results show that the thin CdTeO_3_ alleviates the downward band bending by 40 meV from 470 to 430 meV even though it is consumed during interface formation. The implications of these chemical reactions on the device are assessed through photoluminescence (PL) spectroscopy, which shows losses in internal open circuit voltage (iVoc) from 820 to 795 meV, carrier lifetime from 123 to 45 ns, and PL quantum yield from 2.9 × 10^–5^ to 1.2 × 10^–5^. Modeling time-resolved PL lifetimes demonstrates the back surface recombination velocity due to metallization reduces minority carrier lifetimes. These results identify the native oxide and show that it plays an important role in mediating downward band bending along with how the back interface reaction can negatively impact device-scale parameters and reduce PV PCE.

## Graphical abstract

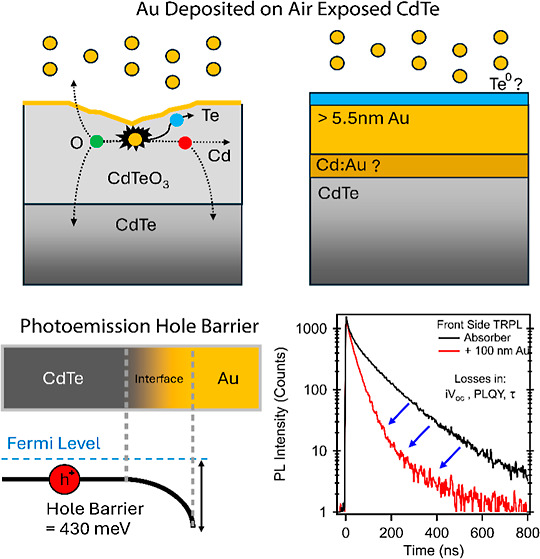

## 1 Introduction

Cadmium telluride (CdTe)-based photovoltaics are the most widely deployed thin-film photovoltaic technology, with roughly 20 GW of global manufacturing capability.^1^ While device efficiencies have advanced recently, the voltage remains persistently low.^2^ The rear, hole-collecting contact has long been scrutinized as a source of this voltage loss and, as the final step of the device fabrication process, can impact more than the rear interface.^3^ This contact is difficult to make due to CdTe’s high density of surface defects^4^ which leads to high recombination velocity.^5^ The desired properties of a back contact to CdTe are interrelated and include favorable valence band alignments minimizing the barrier to holes, a conduction band offset that serves to reflect minority carriers, low interfacial defect density, low hole resistivity, and good chemical stability.^6–8^ This has been achieved in III–V compound semiconductors by methods such as delta doping (including a highly doped buffer layer), alloying between the semiconductor and metal post deposition, and tuning the semiconductor surface to benefit from the chemical reactivity of the deposited metal.^9^

In the case of CdTe, and especially in academic settings, gold is a common back contact material because of its relative ease of use and decent device efficiencies.^10^ The deposition of gold typically follows an air-exposure step, which creates a native oxide of uncertain chemistry^11,12^ and its role in back contacting has been unclear and unexplored. The common practice of exposing partially processed CdTe films to air during device construction has motivated interest in this topic. There is some debate about the exact nature of the CdTe native oxide, with Choi and co-workers suggesting that it is CdTeO_3_ ^12^ and Ponce et al. suggesting that it is TeO_2_ ^11^. The 1981 Ponce work claiming TeO_2_ relied on transmission electron microscopy examination of a sample that had been mechanically polished in cross section and then sputtered with a monatomic ion beam, processes that can severely distort starting material stoichiometry and crystal structure. Recently, the work of Gorai et al. calculated the thermodynamic bulk and interfacial chemical stability of CdTe and (Cd,Te)-containing oxides, suggesting the stable oxides that can form on CdTe are CdO and CdTeO_3_ ^6^. The nature of the CdTe native oxide is important when deciphering its role in contact formation and defects within a device; formation of TeO_2_, for example, from CdTe would imply that a stoichiometric amount of Cd would be generated, which could create interstitials or a surface layer of Cd. In contrast, the formation of CdTeO_3_ would not create Cd^0^ or Cd_i_ because the Cd/Te stoichiometric ratio is equivalent in CdTe and CdTeO_3_. This also has implications for the reaction products when creating an interface with another material.

Drawbacks of CdTe–Au contacts include nonideal band alignment with CdTe,^13–15^ production of a deep energy level defect at 263 meV above the valence band,^16^ high interface recombination, and low device stability. Because CdTe typically has significant band bending and interface recombination at most interfaces, this work reexamines the case of interface formation with a nominally unreactive metal: gold. Chemical reactions are expected to take place in most semiconductor–metal interface formations.^13,17,18^ The general reactions expected between CdTe and elemental metals involve decomposition of CdTe with the formation of another telluride, elemental tellurium and cadmium, and/or a metal–cadmium alloy. As an example, copper was found to react with CdTe(111) to form Cu_2_Te, releasing metallic Cd in the process.^19^ The depositing metal, Cd, and Te can all enter the absorber, creating interstitials, sit at the interface as metal-induced gap states, or diffuse into the metal layer.^13,14^ The reaction that ultimately takes place depends on the relative heats of formation of the CdTe, metal-cation alloy, metal anion (telluride), and pure metal.^13,17,19,20^ A similar process is likely to occur in ZnTe, a common buffer layer for back contacting.^6,13,21^

Previous X-ray photoemission spectroscopy (XPS) work has confirmed that even though bulk gold is a “noble” metal, CdTe undergoes significant chemical changes during CdTe–Au interface formation.^13,14,22,23^ These works did not consider the native oxide, and the barrier measurements were significantly higher than what has been reported in carrier transport measurements.^15^ The observed chemical reactions are likely due to the reactive nature of atomic gold species generated during typical metallization processes.^24^ More reactive metals, such as Mo and Ti, lead to lower open-circuit voltage and device performance, which has been observed in laboratory settings.

If the reaction products stay near the interface, they could generate metal-induced gap states (MIGS) that increase recombination and reduce hole extraction.^13,25–28^ Alternatively, they could diffuse through Au or enter the absorber and create interstitials.^13,14^ Cadmium interstitials (Cd_i_) in p-type CdSe_*x*_Te_1–*x*_ absorbers are double donors; this should increase downward band bending at the back and thereby increase the barrier height.^26,27^ These defects would have midgap defect energy levels that increase recombination and, because they are highly mobile at room temperature, could compensate p-type doping throughout an entire ∼4 μm thick Cd­(Se,Te) absorber layer.^26,27^ Evidence for the metal-induced generation of Cd_i_ is relatively scant, but Wolf and co-workers^29^ used a radio-tracer technique to convincingly show that anomalous diffusion behavior of silver in CdTe was readily explained by cadmium interstitials generated by interfacial reactions occurring between metals and CdTe. Teeter used temperature-programmed reaction in the analysis of reactions between copper and CdTe, directly detecting the evolution of Cd^0^ from the reacting surface.^19^

Additionally, Te^0^ could also diffuse into CdTe at room temperature, creating split Te interstitials (Te_i,split_), which are stable in the neutral and single donor charge states at 0.19 eV above the CdTe valence band.^27^ Finally, when the Au reacts with the CdTe native oxide, oxygen could also be released during the interface formation. Rahman and co-workers have computed O_i_–As_i_–O_i_ to be a donor defect with a negative formation energy in p-type CdTe with a shallow 0.18 eV defect level below the CdTe conduction band and O_Te_–As_Te_ complexes to be acceptor defects.^28^ Thus, all of the metallization reaction products (Cd, Te, and O) could in principle contribute to compensating p-type doping, trapping, and/or recombination.

In this work, the CdTe-native oxide- Au interface is investigated and contextualized in modern devices that utilize air exposure, CdCl2 treatments, selenium, and Group V doping. In Section 2.1, X-ray photoelectron spectroscopy (XPS) is used to identify the CdTe native oxide as CdTeO_3_. Angle-resolved XPS results show that the thickness of CdTeO_3_ on the samples presented here was 1.6 nm. In Section 2.2, the CdTe–CdTeO_3_–Au interface formation is monitored by a stepwise Au deposition, revealing that the CdTeO_3_ layer is consumed via the chemical reactivity of atomic Au, resulting in the final interface being between that of CdTe and gold. In Section 2.3, band positions and hole barriers are measured for CdTe interfaced with CdTeO_3_ and gold using ultraviolet photoelectron spectroscopy (UPS) analysis. When CdTe is interfaced with Au, two schemes are investigated: one in which the native oxide was present prior to Au deposition and the other with the native oxide removed prior to Au deposition, which result in hole barriers of 430 ± 162 meV and 470 ± 162 meV, respectively. The former best represents device processing and is similar to that of carrier transport^15^ and modeling reports.^10^ Although the random error in the absolute value of the measured hole barrier is large, the error in the relative barriers is much smaller (∼42 meV). This is because the relative barrier measurement is purely a calculation of the difference in the (2-) oxidation state of the Te 3d_5/2_ spectra, while the measurement of the magnitude of the barrier height uses UPS, which has an error of 160 meV alone. This demonstrates the native oxide is an interlayer that, while sacrificed in a reaction with gold, partially alleviates the downward band bending stemming from metallization by 40 ± 42 meV. In Section 2.4, the impact of the chemical changes at the back surface on device-scale parameters is investigated by using photoluminescence spectroscopy (PL). PL shows detrimental changes in lifetime, internal open circuit voltage (i*V*_oc_), and PL emission quantum yield. Modeling of carrier lifetimes indicates that the PL has stronger sensitivity to increased back surface recombination velocity than changes in the front/bulk recombination.

## 2 Results and Discussion

### 2.1 Identification of the CdTe Native Oxide

X-ray photoemission spectroscopy (XPS) was performed on the back surface of an air-exposed absorber to understand the sample’s chemical states prior to interface formation (Figure 1a,b). Although these films do incorporate selenium, the selenium content at the back of the absorbers is low. Because of the low sensitivity for selenium with XPS using Al Kα radiation and because of the expected similarity in its chemistry to tellurium, its role in interface formation was not examined in this study. The Te 3d_5/2_ spectrum (Figure 1a) is best fit with three components, which corresponds to Te^2–^ (blue), Te^0^ (red), and Te^4+^ (green). These states represent the Te–Cd (or Te–O in the case of TeO), Te–Te, and Te–O bonding in systems such as CdTeO_3_ and TeO_2_. The peak fitting parameters and results are listed in Table S1. The position of the Te 3d_5/2_ peak for the (4+) oxidation state is well separated from the (2−) and (0) states but has a larger full width at half-maximum (fwhm) reflecting disorder in the compound with a Te (4+) oxidation state. In contrast, the binding energy of the Cd 3d_5/2_ core level has a weak dependence on the chemical environment of the atom^30^ (Figure 1b). The primary peak (blue) is representative of Cd–Te, Cd–Se, and Cd–O bonding, and the higher binding energy peak (yellow) is consistent with Cd–Cl.^31^ The Cl content at the back surface of the films is ∼1% (Table S2); thus, the potential oxidation of CdCl_2_ (Cd oxychloride) contributed very little to the oxygen and Cd signals in this work.

**Figure 1:**
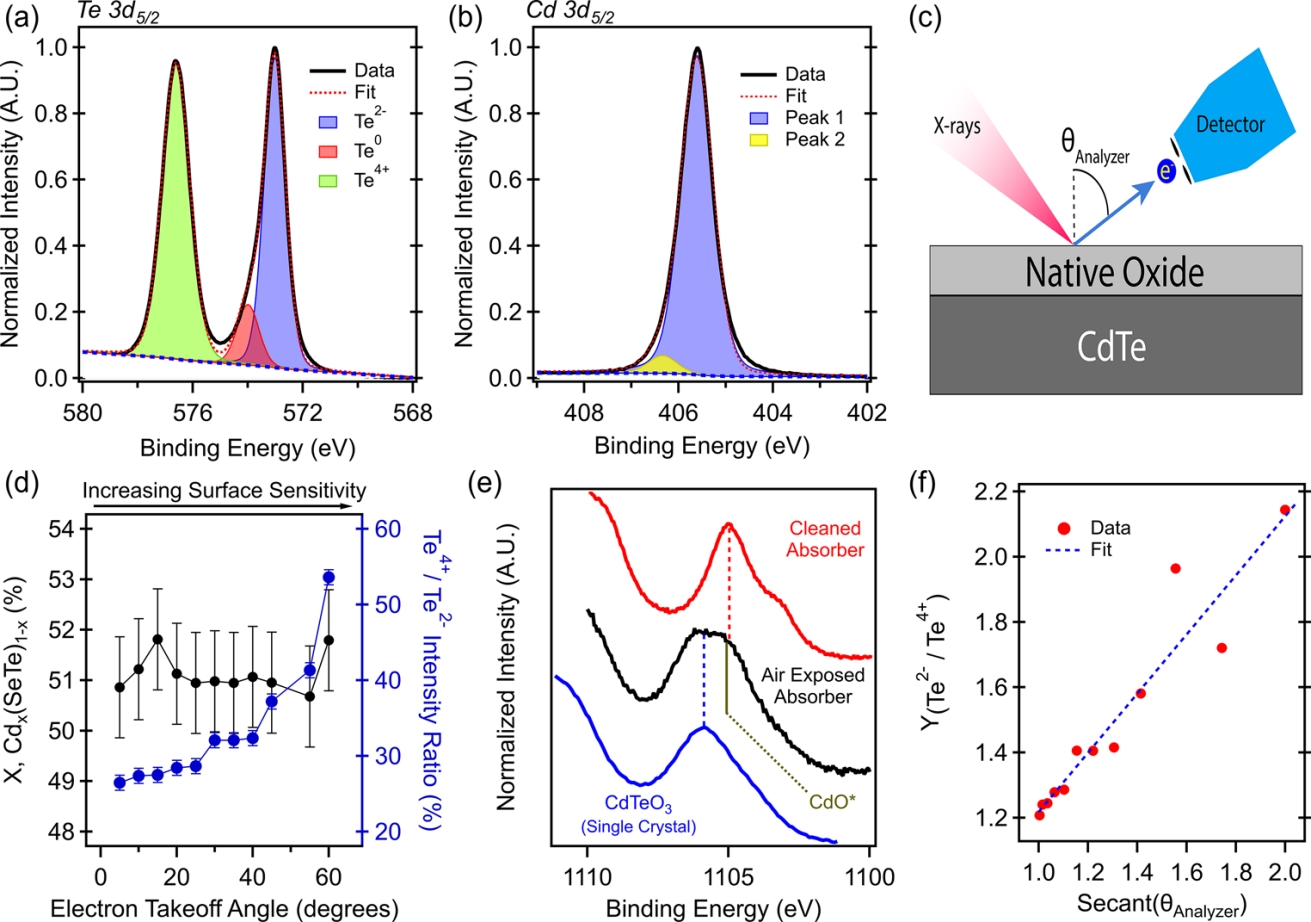
X-ray photoemission spectroscopy results on an air-exposed back surface of CdTe absorbers. (a) Te 3d_5/2_ spectra demonstrating three clear oxidation states and (b) Cd 3d_5/2_ spectra showing a weak dependence on bonding environment, which can be fit with two peaks. In both cases the blue dashed line is the background. (c) Diagram of angle-resolved XPS (AR-XPS). (d) Peak analysis of the AR-XPS. X in Cd_*x*_(SeTe)_1–*x*_ is constant (black data), while the *Te* ^4+^ */Te* ^2–^ ratio climbs (blue data). (e) Cadmium X-ray excited M_4_N_45_N_45_ Auger transition aligned to the Cd 3d_5/2_ core level’s main peak for a cleaned absorber, air-exposed absorber, and CdTeO_3_ single crystal. Vertical red and blue dashed lines show the peak fits for the cleaned absorber and CdTeO_3_, respectively, and the vertical solid green line is the binding energy of the Auger transition for CdO from the Physical Electronics reference database. (f) AR-XPS analysis to determine the thickness of CdTeO_3_ using the Te^2–^ and Te^4+^ oxidation states of the Te 3d_5/2_ spectrum and a calculation of the inelastic mean free path of Te 3d_5/2_ photoelectrons through CdTeO_3_.

To nondestructively investigate the composition of the CdTe native oxide, angle-resolved XPS (AR-XPS) was performed^31,32^ on a chemomechanically polished absorber (Figure 1c,d). The polished absorbers have very similar chemistry to the unpolished absorber (within ∼ 1%) (Figure S1, Table S2, and Table S3) and are flat,^15^ which allows control over the microscopic electron takeoff angle that is not possible on a rough film. The polished absorbers are also polycrystalline, which allows for averaging over the diffraction effects that would occur on a single crystal or epitaxially grown film, which can affect XPS-derived overlayer thicknesses.^33^ As the analyzer tilt is increased, so does the surface sensitivity of the measurement, where the photoelectron takeoff angle = 0° is sample normal.

The tilt-dependence on x in Cd_*x*_(Se,Te)_1–*x*_ and the oxidation state fraction of Te^4+^ to Te^2–^ are plotted in black and blue, respectively, in Figure 1d. With higher surface sensitivity, the amount of Te^4+^ in the XPS signal increases, demonstrating that the native oxide has Te in the (4+) oxidation state in it, which could correspond to CdTeO_3_ or TeO_2_. Along with increasing oxygen contribution to the spectra, the Cd/(Se,Te) ratio remains constant (with an error of 1%). This demonstrates that the native oxide also has Cd in it with the same 1:1 cadmium-to-tellurium ratio as CdTe itself. These results, paired with the thermodynamic stability calculations by Gorai,^6^ suggest that the CdTe native oxide is likely CdTeO_3_ and exclude the possibility that the native oxide is TeO_2_.

As mentioned earlier, the Cd photoelectron lines are not very sensitive to the Cd bonding environment. What can be used instead is the cadmium-modified Auger parameter (AP_Cd_), which is highly sensitive to the cadmium chemical environment^30,34^ (Figure 1e). The value is calculated by the sum of the energies of the Cd M_4_N_45_N_45_ Auger transition in kinetic energy and Cd 3d_5/2_ in binding energy. First, XPS was performed on a gas-cluster ion beam (GCIB)-cleaned CdTe surface, revealing a sharp Cd M_4_N_45_N_45_ Auger transition (Figure 1e, red), and an AP_Cd_ was calculated to be 787.3 ± 0.1 eV, which perfectly matches that of the Physical Electronics database.^30^ Second, a single crystal of CdTeO_3_ was measured (Figure 1e, blue), and its AP_Cd_ was calculated to be 786.1 ± 0.1 eV. The AP_Cd_ for CdO was reported in the Physical Electronics database to be 787.4 eV (Figure S2 and Table S4).

Turning back to the air-exposed absorber, its back surface’s Cd M_4_N_45_N_45_ Auger transition has a flatter-topped Auger transition, which, in its most straightforward explanation, is attributed to two overlapping Cd Auger transitions: one from CdTe and the other from the native oxide. Assuming two chemical components, two maxima were derived from peak fitting using two Voigt functions. The first is 787.3 ± 0.1 eV, which corresponds to that of the cleaned CdTe absorber surface and is also energetically similar to that of CdO (787.4 eV). The second AP_Cd_ value for the air-exposed sample is 785.9 ± 0.1 eV. For comparison, single crystal CdTeO_3_ yields an AP_Cd_ of 786.1 ± 0.1 eV.

The four indications that suggest the CdTe native oxide is CdTeO_3_ are summarized here: (1) Computational work suggested the thermodynamically stable interfaces of CdTe and (Cd,Te)-containing oxides are CdO and CdTeO_3_ ^6^; (2) The native oxide has Te–O bonding in it (in the Te^4+^ oxidation state); (3) The Cd/(Se,Te) ratios of the bulk and native oxide are the same; (4) The AP_Cd_ for air-exposed CdTe was resolved into two components, one of which is close to single crystal CdTeO_3._

The thickness of the CdTeO_3_ on these films was calculated using angle-resolved photoemission analysis.^32^ The effective attenuation length (EAL) of Te 3d_5/2_ photoelectrons energy traveling through CdTeO_3_ was calculated to be 1.4 ± 0.1 nm using National Institute of Standards and Technology (NIST) Standard Reference Database 82 ^35^. First, the tilt dependence of the intensity of the (4+) and (2−) oxidation states of the Te 3d_5/2_ spectra was recorded for the air-exposed polished absorber. Then, reference spectra were taken of UV-O_3_ cleaned single crystal CdTeO_3_ and GCIB-cleaned CdTe to determine the signal strength of the two layers as if they were infinitely thick. These intensities were used to calculate the parameter Y as a function of takeoff angle θ ^31,32^

1 $$Y_{{Te}^{2-}/{Te}^{4+}}\left(\theta \right)=\ln\left\{1+\frac{I_{o}^{A}\left(\theta \right)\cdot I_{CdTe}^{\infty }}{I_{CdTe}^{A}\left(\theta \right)\cdot I_{o}^{\infty }}\right\}=\frac{d_{o}}{L_{o}}\mathrm{Sec}\left(\theta \right)$$

where *I*_o_^A^(θ) and *I*_CdTe_^A^(θ) are the intensities of the air-exposed CdTe’s Te^4+^ and Te^2–^ peaks, respectively, as a function of takeoff angle. Similarly, *I*_o_^∞^and *I*_CdTe_^∞^ are the intensities of the UV-O_3_-cleaned CdTeO_3_’s Te^4+^ peak and GCIB-cleaned CdTe’s Te^2–^ peak, respectively. These are not a function of takeoff because they are pure compounds, and the surface and bulk are chemically equivalent.

The plot of the *Y* parameter as a function of Sec­(θ) (Figure 1f) is expected to be linear until elastic scattering occurs (near θ ∼60° or Sec(60°) ∼2). The measured slope is equal to the thickness of the native oxide multiplied by the EAL. The EAL for photoelectrons with a kinetic energy of 911.56 eV in CdTeO_3_ at a θ = 45° was calculated to be 1.4 ± 0.1 nm^35^. The EAL has a weak dependence on emission angle, which was accounted for in the error bars, but the primary error was in the fit of the *Y* parameter. This measurement and analysis resulted in a native oxide thickness of 1.6 ± 0.2 nm for a film stored and shipped under dry nitrogen but periodically exposed to air during handling. The CdTeO_3_ thickness is expected to increase over time and be dependent on storage conditions.

### 2.2 Analysis of Interface Chemistry During Metallization

To examine the interface between CdTe–CdTeO_3_ and Au, different thicknesses of Au were deposited on the surfaces of air-exposed polished absorbers. At each Au overlayer thickness, the Cd and Te 3d_5/2_ spectra were measured, which can be seen in Figure 2a, with the peaks’ energy shifted such that the (2−) oxidation states are aligned. With increasing Au thickness, the Te^4+^ intensity decreased and was almost absent in the 5.5 nm Au sample, while the Te^2–^ and Te^0^ peaks were still present. The total oxygen signal, measured by the O 1s spectra in Figure S3, nearly disappeared as well, showing the gold released oxygen into either the absorber or the deposition chamber. It also showed the oxide was not converted to another component such as CdO or a gold oxide. If oxygen enters the rest of the device, it may serve as an additional source for oxygen-related defects in the absorber, such as O_Te_–As_Te_ or O_i_–As_i_–O_i_ complexes^28^ or at the front interface, where group V oxides have been reported at high enough levels to be visible in XPS^36^ (which has a lower limit of detection of ∼0.1–1% atomic concentration for most elements).

**Figure 2:**
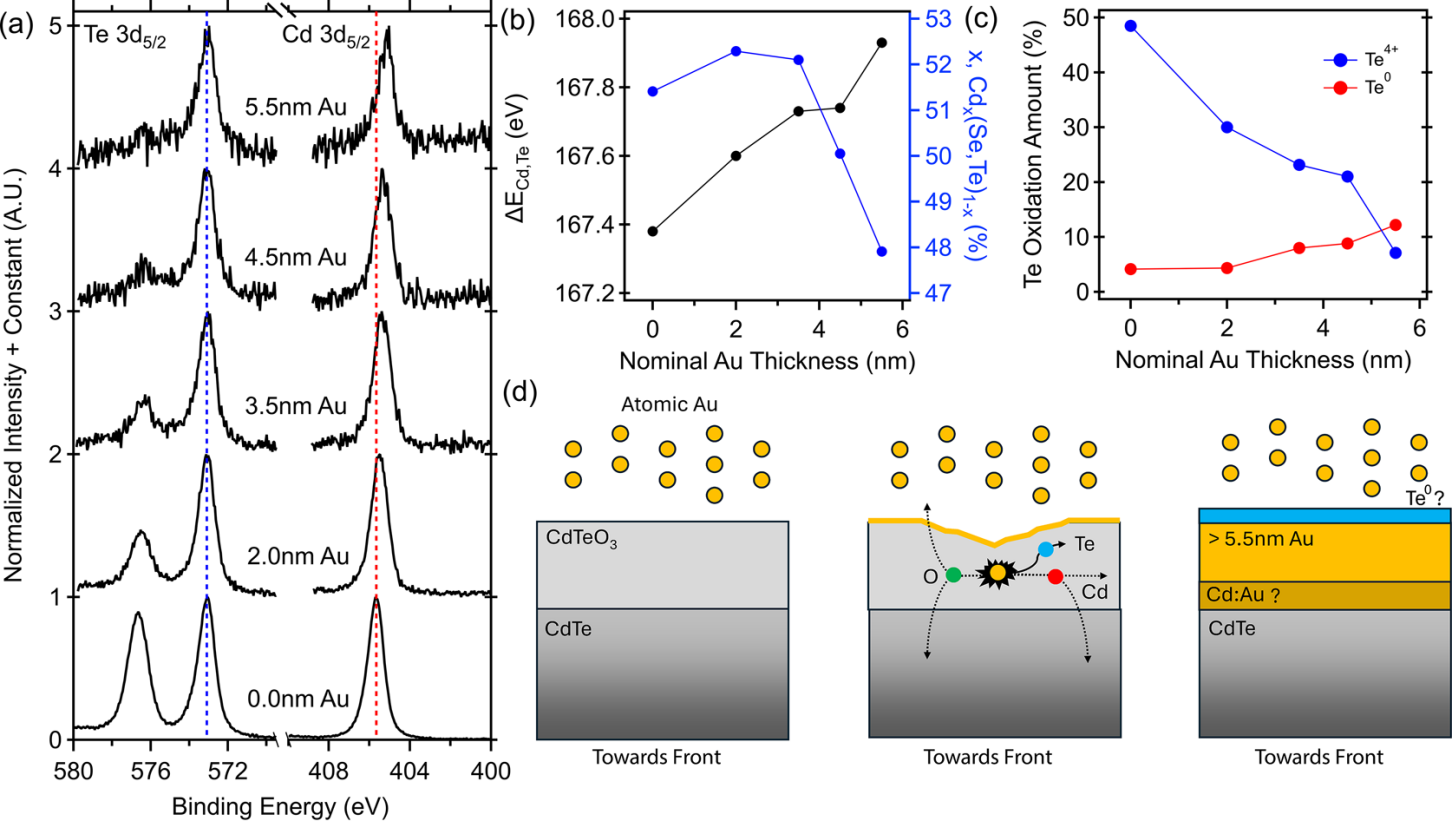
Au overlayer thickness dependent XPS of Te and Cd 3d_5/2_. (a) Energy-referenced spectra to the Te^2–^ peak of the 0.0 nm Au sample (blue dashed overlay). (b) Energy difference between the Cd 3d_5/2_ and Te^2–^ oxidation state of the Te 3d_5/2_ spectrum (Δ*E*_Cd,Te_) and the Cd/Te ratio in percentage as a function of nominal thickness in black and blue, respectively. (c) Percentage of the Te^4+^ (oxide, in blue) and Te^0^ (elemental, in red) within the Te 3d_5/2_ spectrum as a function of nominal thickness. (d) Interface formation diagram based on the XPS results of this experiment paired with the results of Klein and Vitimirov for the Cd/Au alloy and Te^0^ floating on the surface.

The energy separation between the Cd 3d_5/2_ and the (2−) oxidation state of the Te 3d_5/2_ spectra, denoted Δ*E*_Cd,Te_, increased with Au overlayer thickness, which is plotted in Figure 2b in black. By 5.5 nm of Au, the peaks have increased in separation by 550 ± 42 meV. Absolute peak energies of the Cd and Te 3d_5/2_ showed minor energy shifts in the Te^2–^ oxidation state on the order of 10s of meV, which can be attributed to a release of charging or a change in band bending (Figure S4). In contrast, the Cd 3d_5/2_ peak shifts toward lower binding energy on the order of 100s of meV. The differential peak shifts between the Cd and Te 3d_5/2_ imply that a new bonding environment formed, likely in Cd because of its large binding energy changes, but it is unclear what that environment is for Cd in this experiment. Klein and co-workers attributed the binding energy shift, which reached an asymptote with increasing gold thickness, to Cd/Au alloying and Cd_i_ ^13^. The presence of CdTeO_3_ likely changed the thermodynamic energy landscape, which would cause the peak shift asymptote to change.^17^ It could be possible that, given the correct experimental setup and reference spectra, the Cd energy shift asymptote could be observed and fully assigned to a Cd bonding state.

The amount of Cd, relative to Te, within the information depth of the measurement was seen to increase after the first 2 nm of Au and then drop with increasing Au thickness (Figure 2b, blue). The decreasing Cd concentration is consistent with previous work where a total loss of Cd 3d_5/2_ intensity was seen during the clean CdTe–Au interface formation,^13^ which was then used to support the existence of a floating Te layer above the Au layer. The differences seen between the work presented here and Klein et al.^13^ could be differences in final Au-overlayer thicknesses or the sample processing (e.g., the presence of the CdTeO_3_ and/or CdCl_2_).

In the samples studied here, the amount of Te^0^ increased with the Au overlayer thicknesses as the amount of Te^4+^ decreased (Figure 2c). While Te^0^ was clearly produced from Te^4+^ in CdTeO_3_, it may have additionally come from the CdTe because the CdTe–Au interface is likely not abrupt^22,23^ and there is disorder in the CdTeO_3_ (as seen in the enhanced fwhm of the starting Te^4+^ peak), which could allow migration pathways for Au through CdTeO_3_.

The formation of the CdTe–CdTeO_3_-Au interface is chemically complex with CdTe and Au, possibly a Cd–Au alloy, and potentially Au, Cd, Au, and Te at the interface or as interstitials. To assist in understanding, a diagram was built showing the three steps of interface formation (Figure 3d), which was constructed from the results presented here and Klein’s^13^ and Vitomirov’s^14^ results of the Te^0^ at the surface and the Cd/Au alloy (represented by “?” in the diagram). Dashed lines are used to show the possible pathways seen in the measurements. This is also summarized here: Au breaks bonds in CdTeO_3_ such that the oxygen either enters the absorber or is released into the deposition chamber. During this process, there is an overall loss of oxygen from the measurement depth, and the CdTeO_3_ is nearly completely removed by 5.5 nm of Au. Cd and Te are released, which could become interstitial, interfacial, or diffuse in Au. The Cd/(Se,Te) ratio decreases during this process, which converts the Cd-rich absorber into a Te rich interface. Based on the work of Klein and Vitomirov,^13,14^ Te migrates to the surface, where the work presented here confirms that it is in the Te^0^ oxidation state. There are likely other bonding states of Cd that are obscured by peak overlaps, as seen by the continuously increasing binding energy shift in the Cd 3d_5/2_ spectrum. The final interface can be summarized as a Te-rich CdTe-metal interface where the metal is either Au or Au/Cd.

**Figure 3:**
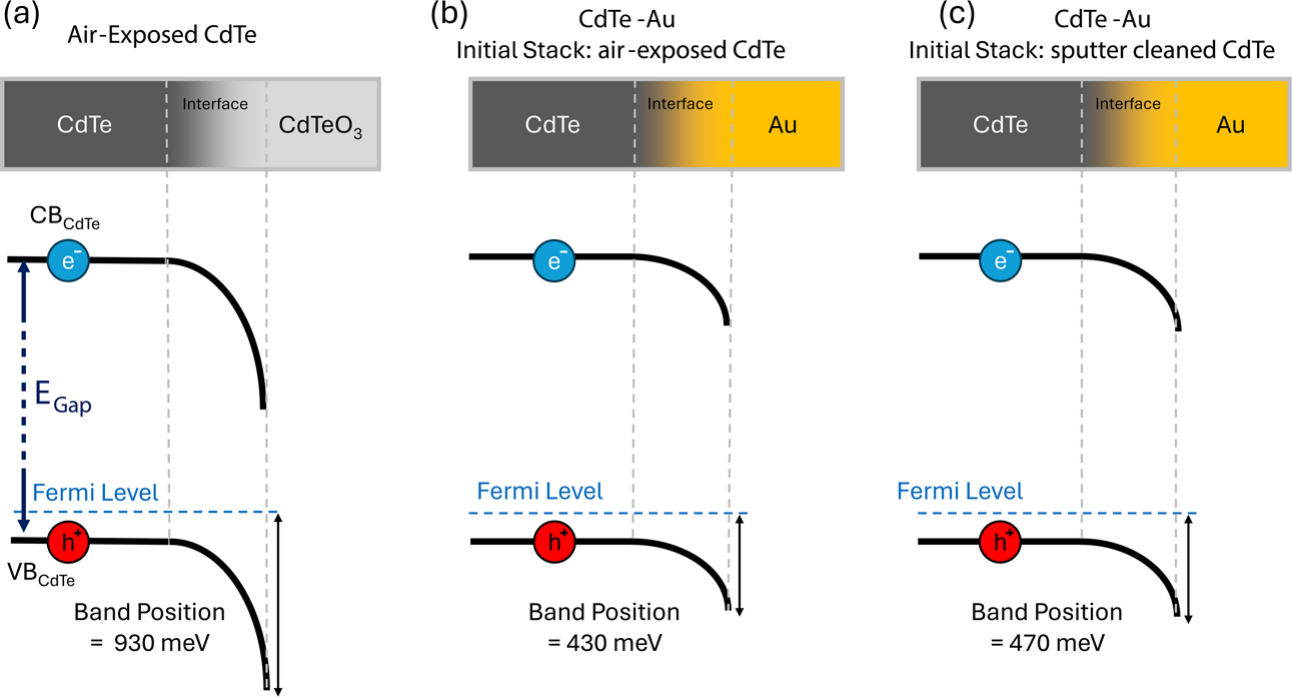
CdTe conduction/valence band (CB_CdTe_/VB_CdTe_) position when interfaced with (a) the native oxide CdTeO_3_, (b) Au with the initial stack being air-exposed CdTe, and (c) Au with the initial stack being GCIB-cleaned CdTe.

### 2.3 Interfacial Band Positions of CdTe

To infer the interfacial valence band position (VB_CdTe,i_) of CdTe underneath the CdTeO_3_ and Au overlayers, the Kraut method was used.^37^ First, a GCIB-cleaned absorber was characterized using ultraviolet and X-ray excitations (UPS and XPS, respectively) to capture the valence band (VB) and core levels of CdTe (Figure S5). This gives the energy difference between the Te^2–^ energy of the Te 3d_5/2_ spectrum and the VB cutoff for CdTe. The presence of elemental cadmium and poor separation between zero valence cadmium (Cd^0^) and Cd^2+^ in CdTe imply that a Te core level is preferred as a proxy for the Cd­(Se,Te) valence band position.^30^ To determine the VB_CdTe,i_ with the overlayers, the Te^2–^ peak position is measured in the air-exposed and Au-coated samples, and then, the VB_CdTe,i_ is inferred using the UPS-XPS reference spectra. The bulk VB position is 180 meV below the Fermi level from the knowledge of doping levels in these films.^21^

In the case of the CdTe–CdTeO_3_ interface, the sample was checked for charging and source-induced photovoltages (Figure S6 and Table S5). VB_CdTe,i_ at the CdTe–CdTeO_3_ interface is determined to be 930 ± 162 meV below the Fermi level with a bulk-to-interface downward band bending of 750 ± 162 meV (Figure 3a). Such a large downward band bending would imply a large hole barrier in a device if the native oxide was remaining at the interface, unless it was thin enough to tunnel through.

In the case of Au overlayers, two samples were created where the first sample was air-exposed prior to metallization (Figure 3b), and the second was GCIB sputter-cleaned to remove the native oxide prior to Au deposition (Figure 3c). The resulting barriers were calculated to be 430 ± 162 meV for the air-exposed and 470 ± 162 meV for the sputter-cleaned samples. The difference is clear in the raw data, and the Fermi-Dirac density of states of the Au overlayer is centered at zero (Figure S7). These VB_CdTe,i_ values are associated with a downward band bending of 250 and 290 meV for the air-exposed and cleaned samples, respectively, showing a 40 ± 42 meV alleviation of the downward band bending after CdTeO_3_ removal. The error of the band alignment measurements is dominated by the UPS valence band cutoff analysis for clean CdTe, which is ±160 meV. But the calculation of the change in band alignment is purely from the change in core levels because the core level to valence band energy difference is assumed to be constant in the Kraut method.^37^ So when calculating the error of the change in band alignment, only the error associated with the core level positions is needed, which is significantly less, ±30 meV.^10^

The alleviation of downward band bending is a demonstration that the native oxide is a beneficial sacrificial layer for Au contacting, partially mitigating the generation of defects during the CdTe−Au interface formation that would increase downward band bending (e.g., donating electrons). Oxygen defects in the O_Te_–As_Te_ complex are acceptors in As-doped CdTe^28^ and could exist below the detection limit of XPS, thus lowering the hole barrier. Alternatively, CdTeO_3_ has less Cd and Te by volume, so the gold reacting with it could produce less Cd and Te per atomic Au deposited. This would reduce the downward band bending by reducing the amounts of Cd_i_ (a fast-diffusing double donor in As-doped CdTe) and Te_i,split_ (neutral or single donors). Other defects could very well be present that drive the partial alleviation of downward band bending. The measurements presented here cannot decipher the underlying mechanism, which governs the reduction of band bending.

The barrier associated with air exposure is similar to temperature-dependent current–voltage measurements, *J* *V*(*T*), where a water-rinsed CdTe–Au device showed a hole barrier of 406 meV.^15^ The measurements here are also similar to that of recent modeling work, which showed a 400 meV hole barrier.^10^ Photoemission spectroscopy measurements reported here spatially average over the back barrier landscape (thousands of grain orientations and boundaries), while transport measurements are dominated by the lower end of the barrier distribution, meaning XPS/UPS measurements will generally report higher barriers than *J* *V*(*T*).^38^

### 2.4 Impacts of Metallization on Voltage in CdTe Solar Cells

Next, voltage losses are compared between samples with a native oxide and with a gold back contact. For this, photoluminescence (PL) spectroscopy was applied to two types of samples: an air-exposed device stack without the back contact (“absorbers”) and one with 100 nm of Au on them (“devices”) (Figure 4a). The back surfaces of the “absorbers” and “devices” are represented by the air-exposed and Au-coated samples studied in the photoemission sections above. The samples were cut from the same absorber stack coupon and stored in the same nitrogen box, so changes from the absorber to the device are attributed to CdTe–Au interface formation. The illumination in PL measurements was through the glass/TCO side of the samples, corresponding to the solar irradiation direction and photon fluence in solar cells.

**Figure 4:**
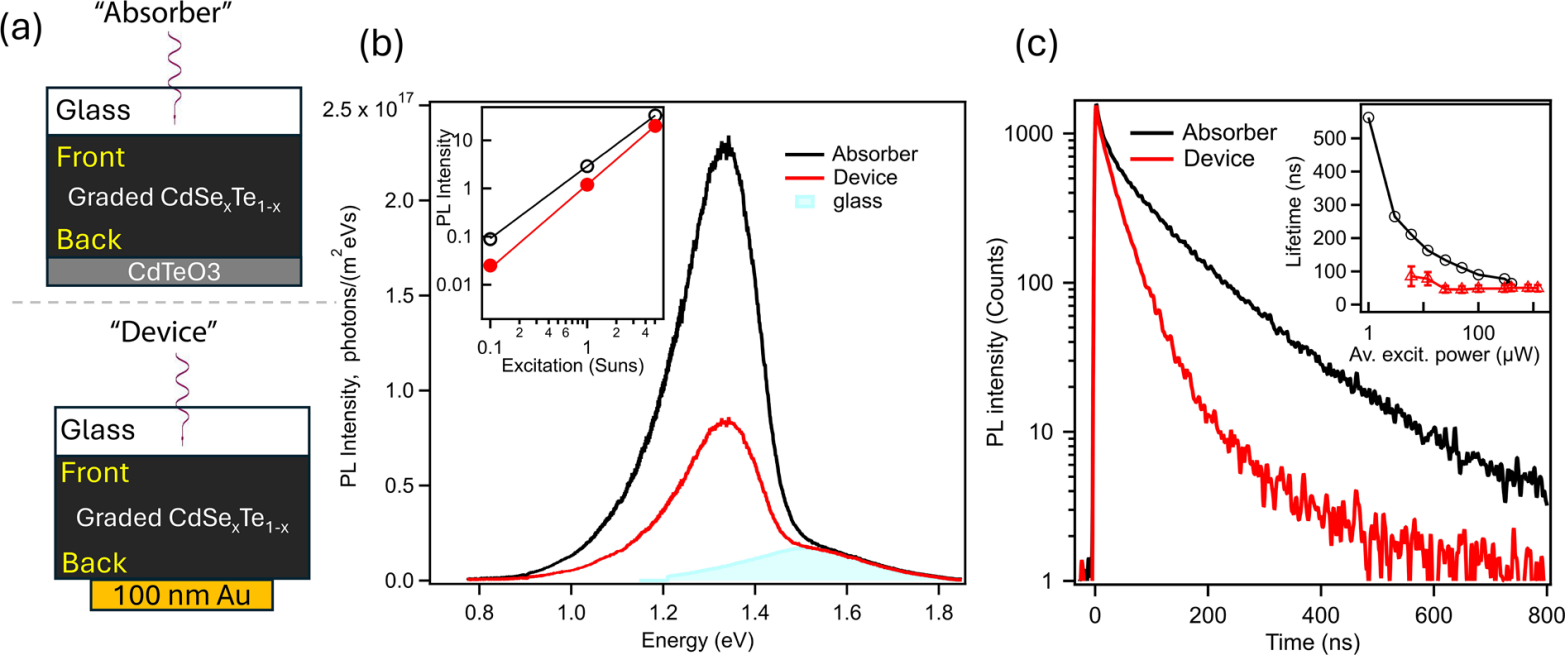
Photoluminescence (PL) data for absorbers and devices. (a) Diagram of sample structures showing that PL measurements were performed on the glass side of samples with and without 100 nm of Au on the back surface. (b) Absolute PL emission spectra measured with 1-sun equivalent excitation. The shaded area indicates scattering from the glass substrate, which was subtracted from the data when estimating radiative efficiencies. The inset shows integrated PL intensity when fluence was 0.1–5.0 suns. The intensity scales with excitation power and is fit using PL Intensity ∼(excitation)^α^. (c) Time-resolved PL (TRPL) data where lifetime τ_2_ is 123 ± 15 ns for the absorber and 45 ± 15 ns for the device. The inset shows the TRPL tail lifetime (fit to 10% of decay) versus the average excitation power.

Figure 4b shows absolute PL emission spectra measured with a 1-Sun equivalent fluence (2 × 10^21^ photons/(m^2^·s)). Broad emission with a maximum below the band gap (here, at *E*_max_ = 1.33 eV) is typical for As-doped Cd­(Se,Te) and is attributed to band tails and hole traps.^39,40^ The peak energies of the two PL emission spectra are equivalent, meaning that there is no observed Stokes shift, which indicates that the absorption and transport gaps are the same between the samples. Within the signal-to-noise for the measurement, PL emission spectra have the same shape between the devices and absorbers, but the intensities (PL emission quantum yields, PLQYs) differ, as summarized in Table 1. Absorptance spectra were calculated from PL emission spectra using the Generalized Planck’s Law and were used to analyze band tail characteristics.^21^ Table 1 also lists band gaps E_g_ (estimated by the absorptance derivative, *E*_g_ = 1.40 ± 0.02 eV) and Urbach energies *E*_u_ = 21.8 ± 0.2 eV (from fits to the absorptance spectra.

**Table 1:** (Photoluminescence (PL) Data Extracted for Samples with and without 100 nm Au on the Back Surface.)^a^

Sample	P*L*_max_ [eV]	PLQY	*E*_g_ [eV]	Stokes Shift [meV]	*E*_u_ [meV]	i*V*_OC_ [mV]	α	τ_2,TRPL_ [ns]
Absorber	1.33 ± 0.01	(2.9 ± 0.03) ×10^–5^	1.40 ± 0.02	70	21.8 ± 0.1	820	1.50 ± 0.01	123 ± 15
Device	1.33 ± 0.01	(1.2 ± 0.02) ×10^–5^	1.40 ± 0.02	70	21.8 ± 0.1	795	1.74 ± 0.01	45 ± 15

a (P*L*_max_ is the energy associated with the maximum of the PL signal. PLQY is the PL emission quantum yield. *E*_g_ is the band gap. *E*_u_ is the Urbach energy. i*V*_oc_ is the internal open circuit voltage. α is the optical diode ideality factor. τ_2,TRPL_ is the second of the exponential fit.).

The detailed balance voltage for this bandgap is 1.22 V,^41^ and band tails of ∼22 meV reduce the voltage entitlement by ∼60 mV^42^ to a radiative voltage *V*_OC_^rad^ = 1.16 V. Using the external yield of radiative emission (PLQY, in the typical range for CdSeTe solar cells^21^), implied voltages (i*V*_oc_) were calculated as ${iV_{OC}=V}_{OC}^{rad}+\frac{k_{B}T}{e}\ln\left(PLQY\right)$ , where $\frac{k_{B}T}{e}$ is thermal voltage. The decrease in PLQY due to metallization is approximately 2.4 times and results in an i*V*_oc_ loss of ∼25 mV (Table 1). The increased slope of PL intensity versus Suns data (inset in Figure 4b) is in agreement with increased Shockley-Read-Hall recombination for the metal-contacted sample.^43,44^ Increased slopes (from 1.50 to 1.74) in PL intensity vs Suns data can indicate increased optical diode factors, which are typically attributed to increased interface recombination.^43,44^

Figure 4c shows TRPL data, which is in general agreement with the stead-state PL. In devices with a junction field, such as solar cells, TRPL decays depend on drift, front junction interface recombination, bulk lifetime (which also depends on grain boundary charges and recombination), and back-contact recombination.^45^ At injection levels where the junction field is screened, the measured rate $\frac{1}{\tau_{TRPL}}$ (τ_TRPL_is TRPL lifetime) can be expressed as a sum of front, bulk, back, and radiative recombination rates^45^

2 $$\frac{1}{\tau_{TRPL}}=\frac{1}{\tau_{front}}+\frac{1}{\tau_{bulk}}+\frac{1}{\tau_{back}}+\frac{1}{\tau_{radiative}}$$

As is commonly used in literature,^45^ the slower component of the TRPL decay was fitted, corresponding to about 10% of the total amplitude and is denoted “τ_TRPL,2_” in the inset of Figure 4c. Lifetime τ_TRPL,2_ more strongly depends on injection (average excitation power) for the absorber (no metallization). For the device, τ_TRPL,2_ = 45 ns, which was approximately constant when injection was varied by 2 orders of magnitude. TRPL lifetime determines the minority carrier (electron) quasi-Fermi level, qFL. Here, the estimated change in *q*FL between the absorber and device $\frac{k_{B}T}{e}\ln\left(\frac{\tau_{TRPL,device}}{\tau_{TRPL,absorber}}\right)$ = $\frac{k_{B}T}{e}\ln\left(\frac{45ns}{123ns}\right)$ ≈ −26 mV is in good agreement with Δi*V*_oc_ = −25 meV from absolute PL data.

Next, the recombination rates, as expressed by eq 2, are analyzed to learn what limits the charge carrier lifetime in the device. The radiative recombination rate, $\frac{1}{\tau_{radiative}}$ = Bp ≈ 10^6^ s^−1^, (where *B* is the radiative recombination coefficient, and *p* is doping) is smaller than the other rates and does not need to be considered. The front interface and bulk recombination rates are grouped together as $R_{front/bulk}=\frac{1}{\tau_{front}}+\frac{1}{\tau_{bulk}}$ . The rate $\frac{1}{\tau_{back}}$ is described using the model of Sproul (valid for an interface with high recombination),^46^ resulting in

3 $$\frac{1}{\tau_{TRPL}}=R_{front/bulk}+1/\left(\frac{W}{S_{back}}+\frac{4}{D}{\left(\frac{W}{\pi }\right)}^{2}\right)$$

where *W* is device thickness, *S*_back_ is back-interface recombination velocity (CdTe–CdTeO_3_ or CdTe–Au), and *D* is carrier diffusion coefficient ( $D=\frac{k_{B}T}{q}\mu$ , where μ is mobility). This expression approximates carrier diffusion (the $\frac{4}{D}{\left(\frac{W}{\pi }\right)}^{2}$ term), back contact recombination (the $\frac{W}{S_{back}}$ term), and front/bulk recombination (the *R*_front/bulk_ term)^47^.

In Figure 5, it is shown how $\frac{1}{\tau_{TRPL}}$ depends on *R*_front/bulk_^–1^, *S*_back_, and μ. Simulated parameter ranges correspond to the literature reports. For example, the lowest reported *S*_back_ for polycrystalline CdTe is 100 cm/s^47^ and the highest value for semiconductor interfaces approaches the thermal velocity of 10^7^ cm/s (e.g., in oxidized GaAs^48^ and for doped Si/aluminum interfaces^49^). For mobility, ∼1 cm^2^/(V·s) was reported due to hole trapping in As-doped CdSeTe,^39^ and mobility was ∼50 cm^2^/(Vs) in As-doped single-crystal CdTe with similar dopant activation.^39^ For lifetimes, Amarasinghe et al. reported 30 to 1300 ns in undoped CdSeTe^50^ depending on composition, and Moseley et al. analyzed lifetimes in this range in As-doped and Se-graded devices.^45^

**Figure 5:**
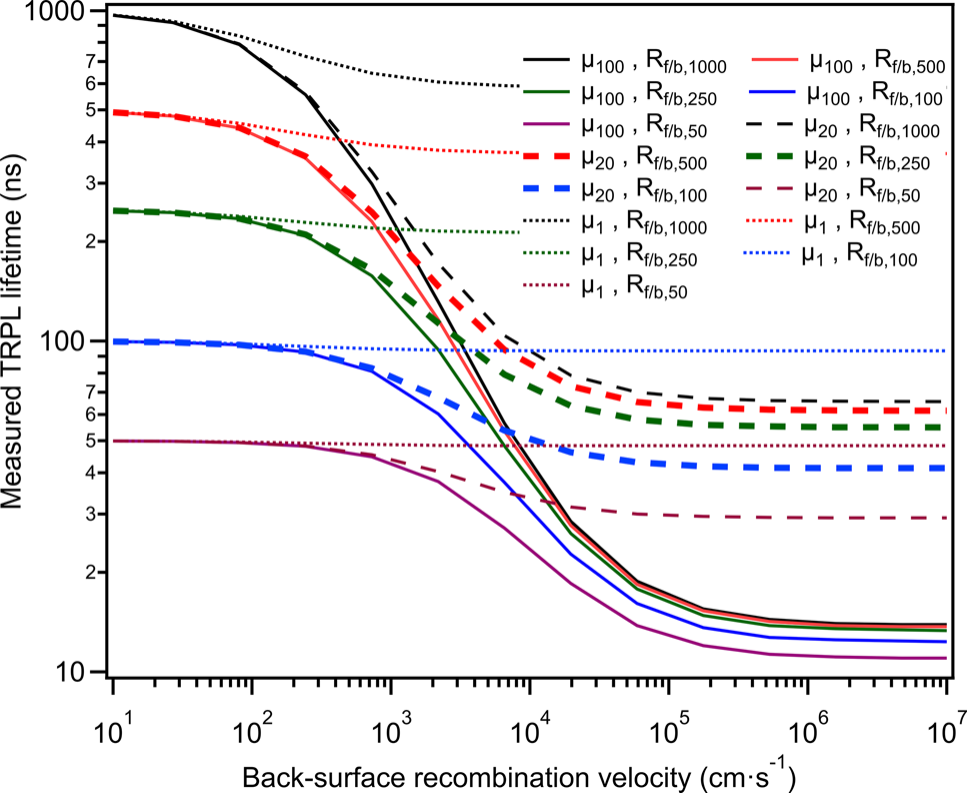
Estimated TRPL lifetimes as a function of back surface recombination velocity, *S*_back_, for a range of mobilities (μ = 100 cm^2^/(Vs), 20 cm^2^/(Vs), and 1 cm^2^/(Vs)) and SRH recombination lifetimes (*R*_front/bulk_^–1^= 1000, 500, 250, 100, and 50 ns). Bold dashed lines show estimates in best correspondence with the experimental data.

While no unique solution for μ, *R*_front/bulk_^–1^, and *S*_back_ can be found from the TRPL lifetime comparisons for this work’s CdTe–CdTeO_3_ and CdTe–Au interfaces, bold dashed lines in Figure 6 indicate the most likely parameter ranges, with bulk and front interface lifetimes *R*_front/bulk_^–1^ = 100 – 500 ns, μ ≈ 20 cm^2^/(Vs), and *S*_back_ ≥ 10^4^ cm/s.

## 3 Conclusion

Here, the CdTe–CdTeO_3_–Au interface is investigated and contextualized in modern devices that utilize selenium alloying and Group V doping. XPS, angle-resolved XPS, and X-ray excited Auger spectroscopy show that the native oxide on CdTe is CdTeO_3_ and not TeO_2_, as has been reported previously.

Evaporated gold, despite being viewed as a “noble” metal, causes extensive chemical changes in the CdTeO_3_. The Cd 3d_5/2_ peak shifts in energy relative to the Te 3d_5/2_ spectrum, which indicates a new Cd chemical state is formed during metallization. By 5.5 nm Au, 1.6 nm CdTeO_3_ that was initially on the sample surface is consumed by the reaction occurring with gold. Oxygen disappears from the back interface and either enters the absorber or is released into the vacuum of the metallization chamber. The Cd/Te ratio at the back interface decreases with increasing Au thickness, suggesting Cd may be entering the absorber. It is also possible that the Te^0^ is floating on the surface of Au, as was shown by previous studies of clean CdTe–Au interfaces being formed in a vacuum.^14,38^

The valence band position of CdTe underneath CdTeO_3_ is determined to be 930 ± 162 meV below the Fermi level, which results from 750 ± 162 meV of downward band bending from the bulk. This suggests that devices that have CdTeO_3_ remaining in the interface can suffer from low hole extraction. However, because gold deposition causes a complete consumption of the native oxide, a band alignment that best represents that of a device is between CdTe and metal.

After gold metallization, hole barriers were reduced relative to those at the free CdTe/CdTeO_3_ interface. The sample with the native oxide on the starting material before metallization resulted in a 430 ± 162 meV hole barrier (250 meV of downward band bending) between CdTe and Au. The second, with the native oxide removed before metallization, resulted in a 470 ± 162 meV hole barrier (290 meV of downward band bending) between CdTe and Au. This shows that CdTeO_3_ is a beneficial native sacrificial layer for direct gold metallization and reduces the CdTe–Au hole barrier by 40 ± 42 meV.

Finally, the effects of the chemical changes in the back interface on the front and bulk of the device are monitored through front-side photoluminescence (PL). The two samples are an air-exposed stack without gold (“absorber”) and one with 100 nm of gold (“device”). The 100 nm gold metallization results in a PLQY reduction from 2.9 × 10^–5^ to 1.2 × 10^–5^, a loss in iVoc from 820 to 795 meV, a change in optical diode ideality factor from 1.50 to 1.74, and finally a loss in τ_2_ lifetime from 123 to 45 ns. These effects are consistent with increased recombination at the CdTe–Au interfaces compared to CdTe–CdTeO_3_. Finally, the TRPL data at an excitation fluence where the junction field is screened suggest stronger sensitivity to increased *S*_back_ than to changes in the front/bulk recombination, which is supported by modeling.

## 4 Experimental Methods

### 4.1 Absorbers and Polishing

All absorber stacks were provided by First Solar, where they, in general, consist of thick soda-lime glass, a transparent conductive oxide stack, and a CdCl_2_-treated, Se-graded, and As-doped Cd­(Se,Te) absorber. A subset of the samples was then polished using a silica slurry. No silica was seen in the absorbers.

Data presented in Figures 1d,f and 2 are for polished absorbers. In the resolution of XPS, the as-received and polished samples’ core levels and valence bands are equivalent. Therefore connections can be directly drawn between the samples (Figure S1, Tables S2 and S3). In general, interface chemistry was observed, and angle-resolved XPS measurements were performed on polished samples to provide a better (flatter) XPS template, while device-scale quantities such as the band alignment and laser-based work were performed on unpolished absorbers.

### 4.2 Gold Depositions

Gold depositions were performed via thermal evaporation in an Angstrom Engineering evaporator at 1 Å/s at room temperature. The evaporator is connected to a nitrogen-filled glovebox to allow for air-free transfers.

### 4.3 Photoemission Spectroscopy

X-ray Photoemission spectroscopy (XPS) experiments were performed using a Physical Electronics Versaprobe III equipped with a hemispherical analyzer under ultrahigh vacuum conditions (∼10^–10^ Torr) at room temperature in a dark chamber to avoid visible light photovoltages. Monochromatic aluminum *K*_α_ photons and, unless otherwise specified, a 5° electron takeoff angle to sample normal were used. For angle-resolved XPS, an aperture was placed in front of the analyzer to reduce the acceptance cone from ±20° to ±5°. A 100 μm X-ray spot size at 100 W was used, which averages over thousands of grain orientations.^15^ The pass energy for Figures 1d,f and 2 is 55 eV, and for Figure 1a,b, it is 27 eV. Pass energy-specific peak fitting procedures were performed with Ulvac-Phi Multipak under best practices outlined by a review paper by Major et al.^51^ Furthermore, we promote following Major and co-workers’ suggested reporting of photoemission spectroscopy instrumental/fitting metadata.^52^

Ultraviolet photoemission spectroscopy (UPS) was performed on the same instrument using a He source without a monochromator. To remove the He_α, β, γ_ satellite peaks, a procedure was developed using the Fermi edge feature observed on clean molybdenum foil. A pass energy of 6.5 eV was used, and dual XPS-UPS measurements were performed to account for UV-induced photovoltages.

An argon gas-cluster ion beam (GCIB) sputter tool was used for cleaning to determine constraints for peak fitting of the air-exposed and gold-coated samples. For this, the beam energy and current were 15 kV and 35 nA, respectively, with a 2 × 2 mm^2^ raster. Gas clusters were nominally arranged in Ar_2000_^+^. GCIB-cleaned gold and copper foils were used to calibrate the energy scale of the analyzer (Figure S8 and Table S6).

Post deposition, the glass/TCO/Cd­(Se,Te) film stacks were electrically contacted by exposing the transparent conductive oxide (TCO) on the edges of the samples. Then carbon tape was placed on the TCO, absorber, and gold (if present), which was then attached to the XPS platen. The mounted samples were transferred into the XPS chamber without exposure to air by using a physical electronics model 07–111 K transfer vessel. This is necessary because exposing the Au-coated samples to air will cause a significant amount of oxidation of the underlying CdTe, which was seen in a control sample measured without and with air exposure (Figure S9).

### 4.4 Photoluminescence Spectroscopy

Photoluminescence (PL) spectroscopy was performed using a 632.8 nm excitation (HeNe laser) using an *f* = 300 mm spectrograph with calibrated Si and InGaAs detectors (Princeton Instruments). For the absolute intensity calibration, a 2% reflectance standard was used (LabSphere).

Time-resolved PL (TRPL) decays were measured with excitation at 640 nm (300 fs pulses at 330 kHz repetition rate, Pharos/Orpheus OPA system, Light Conversion). Detection for TRPL was at 920 nm (1.35 eV) with a Si avalanche photodiode, and time-correlated single photon counting (with Picoharp 300 electronics, Picoquant) was used.

### 4.5 Growth and Characterization of the CdTeO_3_ Crystal

Single crystal CdTeO_3_ was grown by using a modified Brigman technique. Two grade-5 powders (CdO and TeO_2_) with equal molar percentages were first mixed using a Stoneware jar mill for a total milling time of at least 15 h. The resulting mixture was then pressed using a hydrostatic press at 20,000 psi. After pressing, the powder clump was placed in a box furnace and calcined at 625 °C. This sequence of mixing, pressing, and calcining was applied to all mixtures and was intended to promote the formation of a stable solid-state solution.

To grow the crystal, a customized hot chamber constructed from insulating materials was built around the crucible. The design emphasizes the creation of a temperature gradient to mimic Bridgman crystal growth.

The CdO–TeO_2_ calcined lump was placed in a dish-shaped platinum crucible, which was then covered with a platinum disk to stabilize the melt and reduce the loss of volatile CdO. The assembly was heated in an RF furnace. After melting was achieved, the temperature was increased by 20–40 °C and then held at that temperature for 1 h to allow the melt to stabilize. Following that, the temperature of the assembly was lowered by 1 °C per hour until all the melt had crystallized below the melting temperature by about 30 °C. The assembly then was brought down to room temperature at 10 °C/h per hour.

The crystal was further confirmed to be CdTeO_3_ by Raman spectroscopy and powder X-ray diffraction (XRD) (Figure S10). For Raman spectroscopy, 632.8 nm excitation was generated by using a HeNe laser. The focus was set to 20x with an ND filter set such that the nominal power is 7 mW. The analyzer was calibrated using Si, and a diffraction grating of 1800 lines/mm was used. The XRD sample was prepared by hand-grinding a crystal in a quartz mortar and mounting the powder on a glass slide with adhesive tape. XRD measurements were performed using a Rigaku SmartLab instrument (Rigaku Corporation, Akishima, Tokyo, Japan) with a rotating anode (45 kV, 200 mA), Cu K_α_ radiation (Cu K_β_ filter installed), and a HyPix-3000 detector. θ–2θ scans from 8° to 90° were performed in the Bragg–Brentano geometry in one-dimensional detector mode using a continuous scan with a 0.02° step interval, a scanning speed of 4°/min, and an incident slit angle of 0.5°.

Absorption spectroscopy was performed using a DT 1000 CE UV/vis light source and OOIBase 32 Spectrometer Operating Software from Ocean Optics. Samples were polished to ensure parallel surfaces and then positioned perpendicular to the light source, with transmitted light collected by a detector. Calibration included recording the dark and reference spectra prior to measurements. The resulting spectra were used to estimate the optical band gap. The band gap was determined to be 3.9 eV (Figure S10b), matching well with the theoretical prediction of 3.91 eV.^53^

## Supplementary Material



## Data Availability

The data associated with this study will be openly available in the NLR data catalog at https://doi.org/10.7799/3020858.

## Notes

Fitting parameters of X-ray photoemission spectroscopy (XPS) for air-exposed CdTe and sputter-cleaned unpolished and polished absorbers, experimental and reference cadmium modified Auger parameters (AP_Cd_), O 1s spectra before and during metallization, raw Cd and Te 3d_5/2_ spectra during CdTe–CdTeO_3_–Au interface formation, ultraviolet photoemission spectroscopy (UPS) and XPS used to determine the band position of CdTe, photoelectron charging effects of air-exposed CdTe, calibration of the photoemission detector, Au-coated CdTe air exposure results, and CdTeO_3_ photograph, Raman spectroscopy, powder X-ray diffraction (XRD), and absorbance spectroscopy (PDF)
