# Supplementary material for: Gold-Induced Chemical Perturbations in CdTe-Based Photovoltaic Cells
Source: ACS Appl Mater Interfaces. 2026 Mar 20;18(12):18417–27. doi: 10.1021/acsami.5c24315 (PMC13051422; doi:10.1021/acsami.5c24315)
Supplement: Supplementary file 1 [file am5c24315_si_001.pdf]

Supporting Information

Gold-Induced Chemical Perturbations in CdTe-Based Photovoltaic Cells

Ryan Muzzio[1,], Darius Kuciauskas[1], B. Edward Sartor[1], Joshua A. Brown[1], Jalal Nawash[2], Joel N. Duenow[1], Hongling Lott[1], Chungho Lee[3], Matthew O. Reese[1], and Craig L. Perkins[1]*

1 National Laboratory of the Rockies (NLR), Golden, Colorado 80401 USA

2 School of Mechanical and Materials Engineering, Washington State University, Pullman, Washington 99164, USA

3 First Solar Inc., California Technology Center (CTC), Santa Clara, California 95050, USA

*Corresponding Author

E-Mail: Ryan.Muzzio@NLR.gov

Table of content:

1. **X-ray photoemission spectroscopy (XPS) fit values for air exposed CdTe**
   (Table S1) Page S3
2. **XPS comparison of the unpolished and polished absorbers**
   (Figure S1, Table S2, Table S3) Page S3-4
3. **Experimental and reference cadmium modified Auger parameters**
   (Figure S2, Table S4) Page S5
4. **Total oxygen signal during Au metallization**
   (Figure S3) Page S6
5. **Raw Cd $3d_{5/2}$ and Te $3d_{5/2}$ spectra during Au overlayer thickness series**
   (Figure S4) Page S6
6. **Determining the Te^{2-} to valence band position using XPS and ultraviolet photoemission spectroscopy (UPS)**
   (Figure S5) Page S7
7. **Charging in the CdTe-$CdTeO_3$ interface**
   (Figure S6, Table S5) Page S8
8. **Raw XPS data used to determine hole barriers in CdTe-Au samples**
   (Figure S7) Page S9
9. **Determining the linearity of the detector**
   (Figure S8, Table S6) Page S9-10
10. **2 nm Au overlayer on CdTe with and without air exposure**
    (Figure S9) Page S10
11. **Single crystal $CdTeO_3$ structural and absorbance characterization**
    (Figure S10) Page S11

References Page S12

1. X-ray photoemission spectroscopy (XPS) fit values for air exposed CdTe

Peak	Binding Energy [eV]	FWHM [eV]	Percent of Spectral Region [%]
Te^{4+}	576.6	1.19	51 %
Te^{0}	573.96	0.94	8 %
Te^{2-}	573.01	0.92	41 %
$Cd_{peak\ 2}$	406.37	0.81	95 %
$Cd_{peak\ 1}$	405.61	0.72	5 %

Table S1: Quantitative peak fitting results of Figure 1 a,b. All fit functions were Voigts with 85% gaussians with Shirley backgrounds

2. XPS comparison of the unpolished and polished absorbers

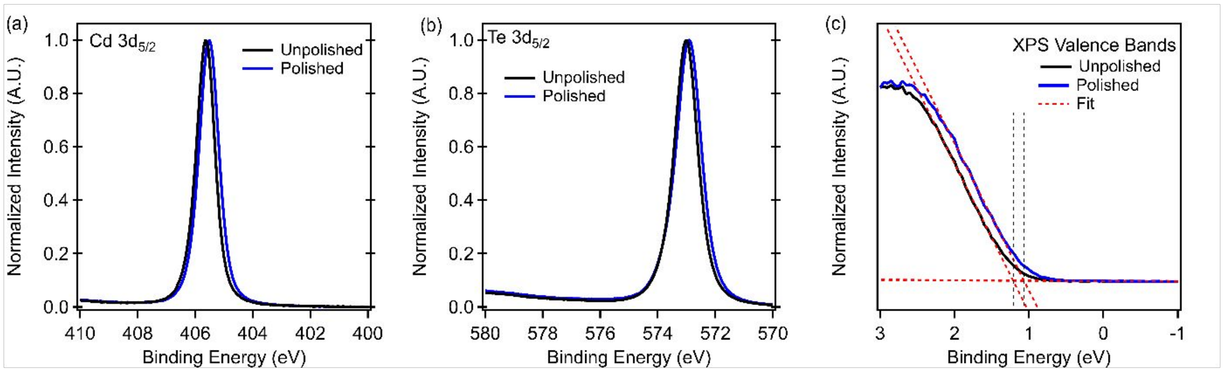

Figure S1: Raw x-ray photoemission spectroscopy data of the GCIB cleaned unpolished and polished absorbers' (a) Cd $3d_{5/2}$ peaks (b) Te $3d_{5/2}$ peaks and (c) valence bands. Upon analysis, it is shown that the spectra are rigidly shifted (see Table S3). This is demonstrated by $E_{VB(Te)}$ and $E_{VB(Cd)}$ which are the energy differences between the valence band (E_{VB}) and the Te^{2-} and $Cd_{Peak\ 1}$ which only changes by 40 meV and 50 meV respectively between the absorbers. The differences seen here are within the error of the XPS measurements.

(**Table S2**: Atomic concentrations of the unpolished and polished absorber. Labels follow Figure 1 of the main text)

Sample	X, $Cd_x(Se,Te)_{1-x}$ [%]	$Cd_{peak 1}$ [%]	$Cd_{peak 2}$ [%]	Te^{2-} [%]	Te^0 [%]	Cd(Se,Te) [%]	O [%]	Cl [%]
Standard	52.1	96.45	3.55	96.36	3.64	66	33	1
Polished	51.1	95.61	4.39	94.92	5.08	67	33	0

(**Table S3**: Fit parameters related to the energies of the unpolished and polished absorbers)

Sample	$Cd_{peak 1}$ [eV]	$Cd_{peak 2}$ [eV]	Te^{2-} [eV]	Te^0 [eV]	E_{VB} [eV]	$E_{VB(Te)}$ [eV]	$E_{VB(Cd)}$ [eV]	$\Delta E_{Cd,Te}$ [eV]
Unpolished	405.66	406.46	573.00	574.03	1.21	571.79	404.44	167.34
Polished	405.54	406.28	572.91	573.89	1.07	571.84	404.47	167.37

(All peaks are labeled under the same labeling scheme as Figure 1 a,b in the main text. E_{VB} is the valence band energy, $E_{VB(Te)}$ and $E_{VB(Cd)}$ are the energy differences between the valence band and Te $3d_{5/2}$ Te^{2-} oxidation state and the Cd $3d_{5/2}$ main peak respectively, and $\Delta E_{Cd,Te}$ is defined in the main text as the energy difference between Te $3d_{5/2}$ Te^{2-} oxidation state and the Cd $3d_{5/2}$ peak 1)

3. Experimental and reference cadmium modified Auger parameters

Experimental values from this work for the Cd Auger Parameter ($AP_{Cd,Exp}$) and reference data ($AP_{Cd,Ref}$) from the Physical Electronics Handbook [1]. The Auger transition and background shape differences complicate the analysis of samples in which Cd is bonded to more than one type of atom (air-exposed CdTe).

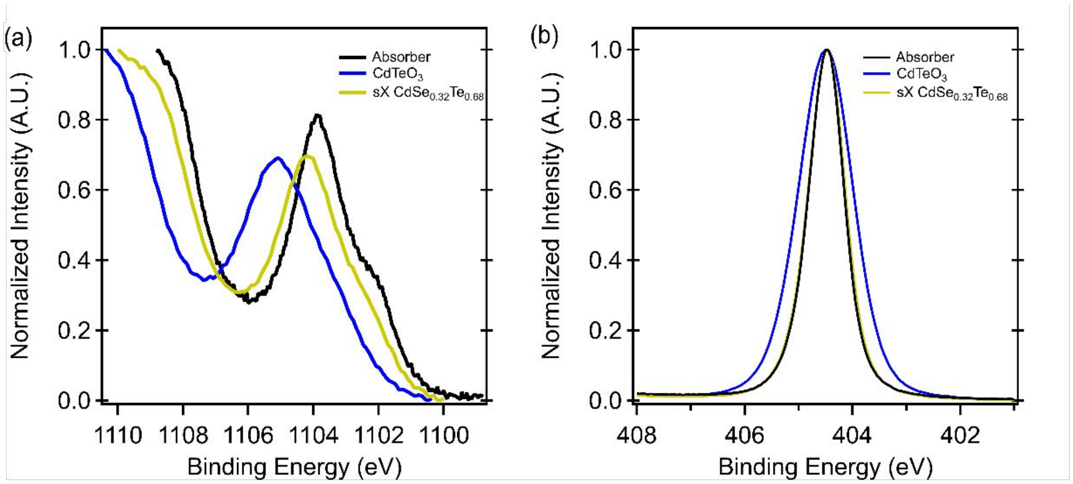

Figure S2: Cadmium modified Auger parameters for various samples. (a) the Cd $M_4N_{45}N_{45}$ Auger transition. (b) the Cd 3d 5/2. All spectra are energy referenced to the Cd 3d5/2.

(**Table S4**: Quantitative results of experimental and reference cadmium modified Auger parameters labeled as $AP_{Cd,Exp}$ and $AP_{Cd,Ref}$ respectively. Quantifying the error on these peak fitting procedures is difficult because of the complicated Auger line-shape and background that depend on chemical state, but an estimated peak position uncertainty of ± 0.1 eV was assigned to the measurements. All $AP_{Cd,Ref}$ are from the Physical Electronics handbook of XPS [1] except for $CdTeO_3$ which is from the NIST database [2])

Sample	$AP_{Cd,Exp}$ [eV]	$AP_{Cd,Ref}$ [eV]
Absorber	787.3	---
CdTe	787.3	787.3
$CdTeO_3$	786.1	785.8

CdO	---	787.4
$CdSe_{0.32}Te_{0.68}$	787.0	---
CdSe	---	786.7

4. Total oxygen signal during Au metallization

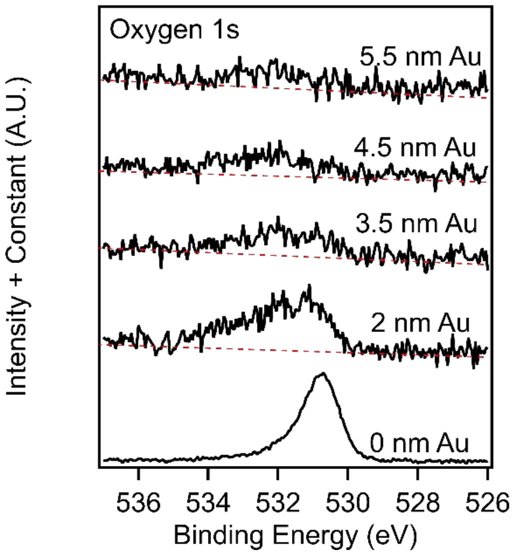

Figure S3: The total oxygen signal, monitored through the O 1s core level, decreases with increasing Au overlayer thickness. It is unclear if the oxygen is escaping into the deposition chamber or being sequestered into the absorber. All scans were taken with a 55 eV pass energy except for the 0 nm Au sample which was taken with 27 eV pass energy. If it were taken with a 55 eV pass energy the peak energy and shape would not change.

5. Raw Cd $3d_{5/2}$ and Te $3d_{5/2}$ spectra during Au overlayer thickness series

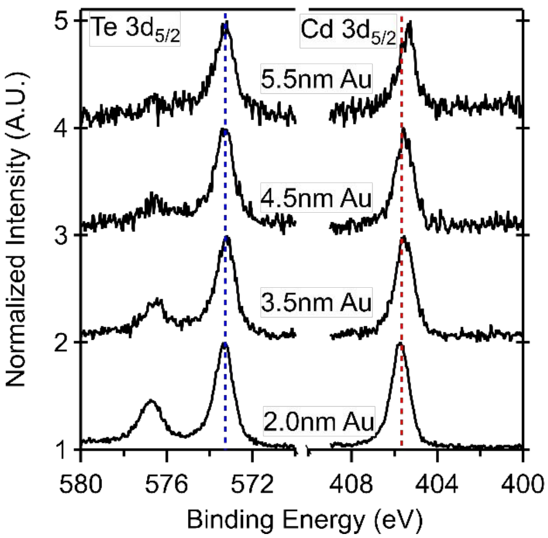

Figure S4: Absolute peak energy spectra of the Cd $3d_{5/2}$ and Te $3d_{5/2}$ during metallization. The raw data highlights Cd $3d_{5/2}$ moving to lower binding energies. The dashed lines show where the peaks should be for a $\Delta E_{Cd,Te}$ = 0 eV. Figure 2 of the main text has the spectra aligned to the (2-) oxidation state which is useful to compare the metallized spectra to that of a bare absorber, which has severe downward band bending.

6. Determining the Te^{2-} to valence band position using XPS and ultraviolet photoemission spectroscopy (UPS)

To determine the energy difference between the Te^{2-} oxidation state of the Te $3d_{5/2}$ and the valence band of the absorber, an unpolished absorber was cleaned using a gas-cluster ion beam. Then, ultraviolet photoemission spectroscopy (UPS) was used to probe the valence band. The valence band cutoff was determined by performing a helium satellite removal procedure. This procedure was developed under the same experimental conditions but with a Mo foil as a sample to measure the Fermi-Dirac feature at 0.0 eV. The data was plotted in a semi-log format and then fits (red dashed line) were performed at the linear intensity and background levels. This technique which has been shown to reveal valence band alignments that align with modeling [3] [4]. The result is shown as the blue dashed line. The Cd and Te $3d_{5/2}$ are then measured under UV and X-ray light exposure. This accounts for any photovoltage due to the UV light which then makes the binding energy axes equivalent for all plots. The peak fits are shown in the top right for the core levels. The band position of CdTe at the two interfaces is then measured by the Kraut method [5].

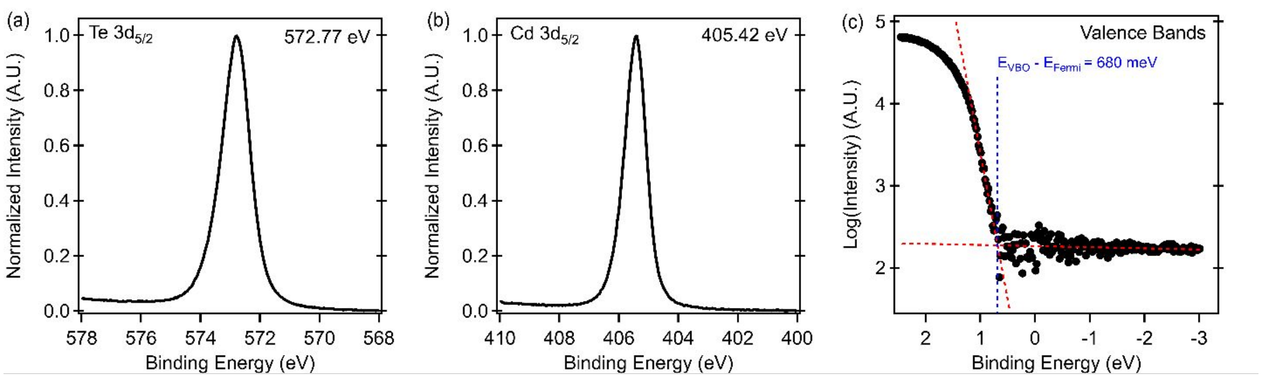

Figure S5: Photoemission spectroscopy performed to use the Kraut method described in the main text section 2.3. (a,b) Te $3d_{5/2}$ and Cd $3d_{5/2}$ respectively with excitations from XPS and UPS. (c) UPS valence band spectra.

7. Charging in the CdTe-$CdTeO_3$ interface

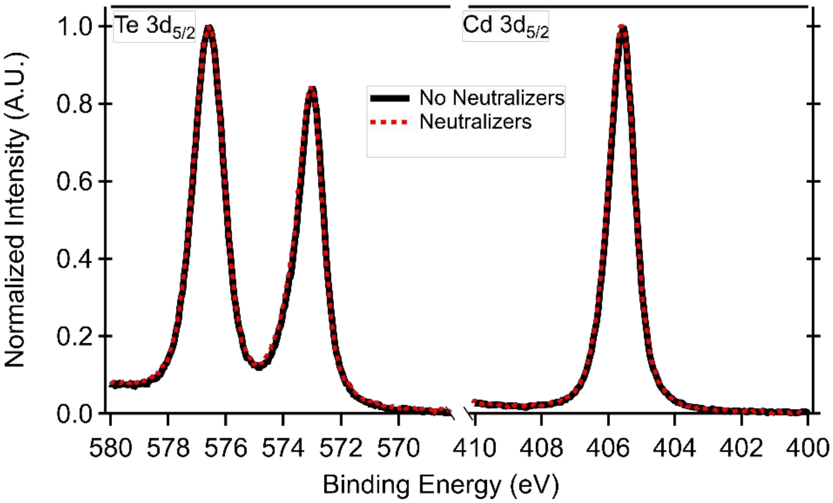

Figure S6: Dual electron and hole neutralizers are used to check for charging in the $CdTeO_3$-CdTe sample. These neutralizers will put electrons and holes on to the sample surface to remove charging due to the photoemission process [6]. The charging was only

seen in the Te^{4+} oxidation state (100 meV), but the Te^{2-} is used to calculate the band position.

(**Table S5**: Quantitative peak analysis of Figure S6).

Sample	Te^{2-} [eV]	Te^{0} [eV]	Te^{4+} [eV]	$Cd_{Peak\ 1}$ [eV]	$Cd_{Peak\ 2}$ [eV]
No Neutralizers	573.02	574.05	576.59	405.51	406.28
Neutralizers	572.96	574.01	576.49	405.51	406.28
ΔE	0.06	0.04	0.1	0	0

8. Raw XPS data used to determine hole barriers in CdTe-Au samples

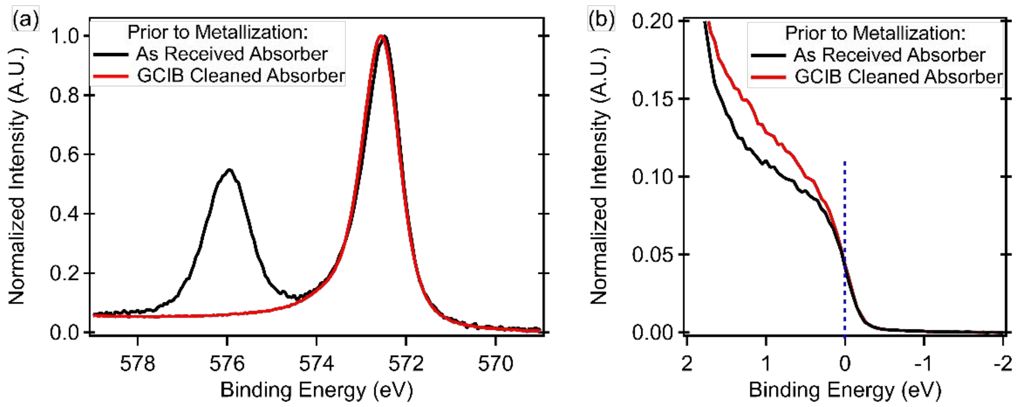

Figure S7: Raw XPS data of the Te $3d_{5/2}$ and the valence bands. (a) The air exposed and sputter cleaned Te $3d_{5/2}$ in black and red respectively. The air-exposed sample has a Te^{2-} peak closer to the Fermi level than the cleaned sample. Fit values of the Te^{2-} oxidation state of the Te $3d_{5/2}$ are 572.52 and 572.56 eV for the air-exposed and cleaned samples. (b) The x-ray excited valence bands of the CdTe-Au interface. A clear Fermi

Dirac distribution is seen from the Au overlayer. Without adjustments, both Fermi Dirac inflection points are centered at zero as expected.

9. Determining the linearity of the detector

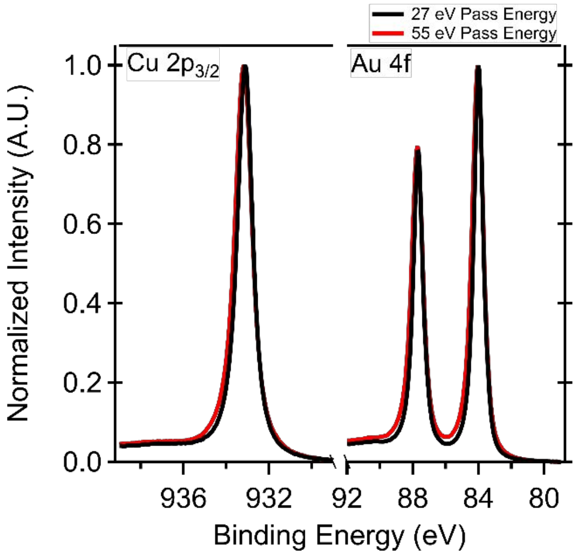

Figure S8: XPS data of clean Cu and Au films. The linearity of the detector is checked and confirmed to be invariant of the pass energy. This is performed by measuring and analyzing the peaks of low and high energy core levels such as the Au 4f and Cu $3p_{3/2}$ respectively (left). The peak fits are shown in the table with the reference spectra taken from Physical Electronics' Handbook of X-Ray Photoemission Spectroscopy [1]. Quantitative results in Table S6).

(**Table S6**: quantitative analysis of Figure S8.)

Measurement	Cu $2p_{3/2}$ [eV]	Au $4fp_{7/2}$ [eV]	Au $4fp_{5/2}$ [eV]
27 Pass Energy	933.11	84.01	87.68
55 Pass Energy	933.18	84.06	87.74
Reference	933	84	88

10. 2 nm Au overlayer on CdTe with and without air exposure

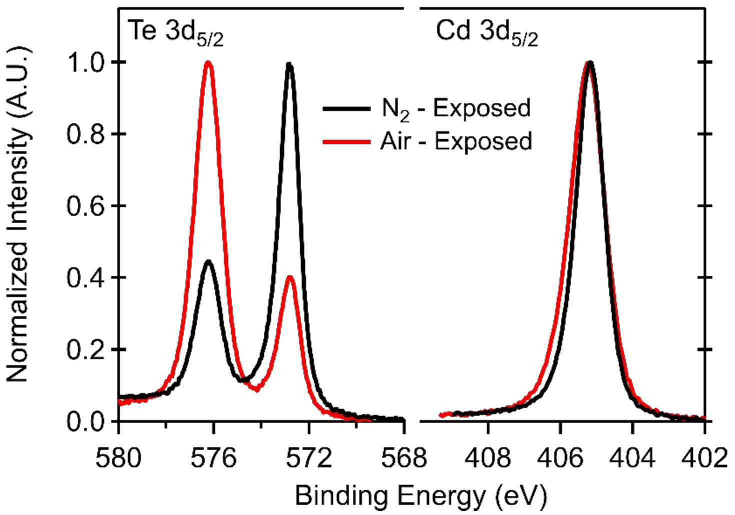

Figure S9: XPS of the Te and Cd $3d_{5/2}$ before and after air exposure. The N_2-exposed sample had only been in vacuum and N_2 before measurement (black). The sample was then left in the lab environment for 16 hours and re-measured which showed a significant increase in the Te^{4+} peak (red).

11. Single crystal $CdTeO_3$ structural and absorbance characterization

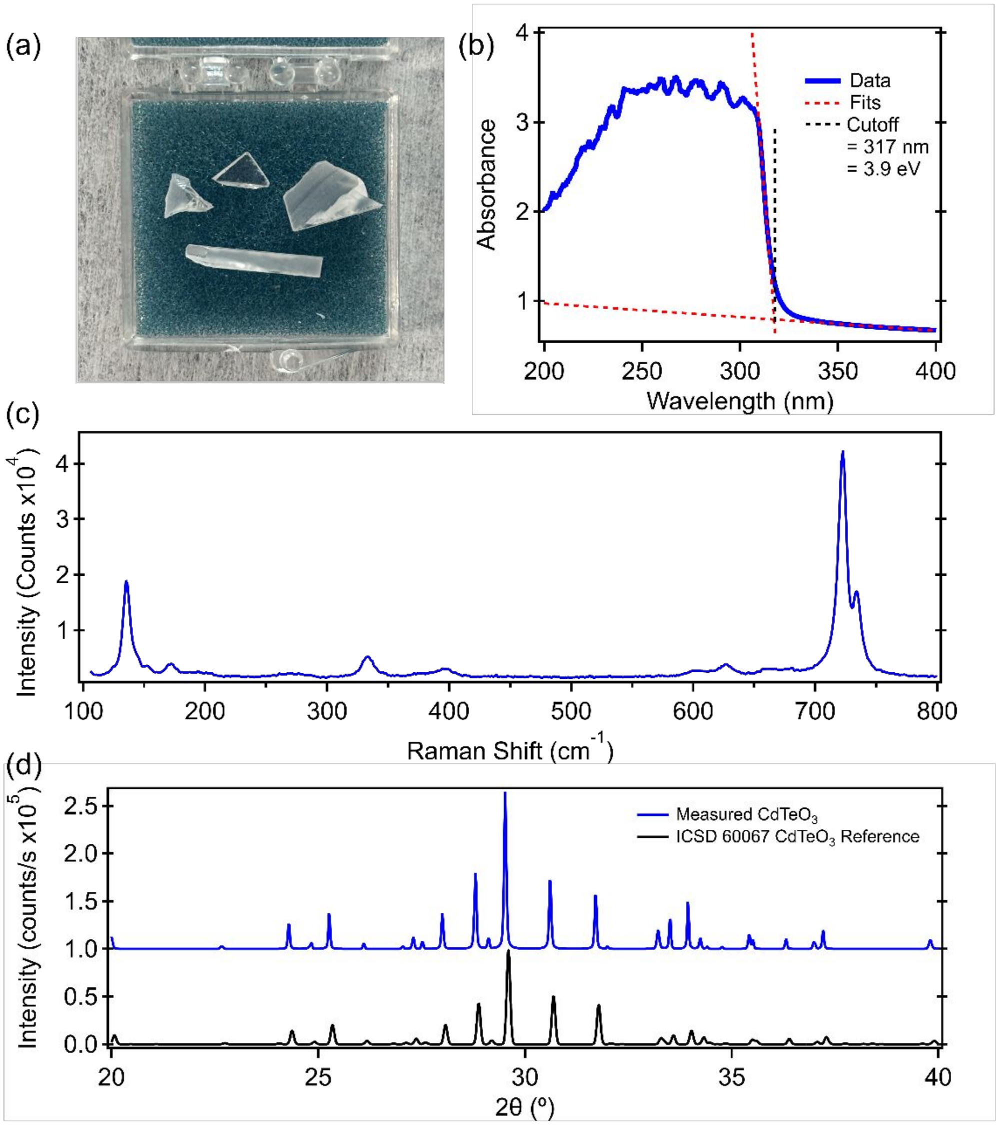

Figure S10: Photograph and structural characterization of single crystal $CdTeO_3$. (a) photograph of the single crystals. (b) Absorbance spectroscopy which was used to measure the optical band gap to be 3.9 eV which closely matches a theoretical prediction of 3.91 eV [7]. (c) Raman Spectroscopy which closely matches spectra from a high purity $CdTeO_3$ powder [8]. (d) Powder x-ray diffraction and comparison to a reference spectrum [9].

References:

[1] J. Chastain and R. C. King Jr., *Handbook of X-ray photoelectron spectroscopy*. Perkin-Elmer Corporation, 1992.

[2] C. D. Wagner, A. V. Naumkin, A. Kraut-Vass, J. W. Allison, C. J. Powell, and J. R. Jr. Rumble, "NIST Standard Reference Database 20." 2003. [Online]. Available: http:/srdata.nist.gov/xps/

[3] M. Nardone *et al.*, "Comprehensive model for evaluating voltage losses and performance improvements in thin-film photovoltaic devices," *Phys. Rev. Appl.*, vol. 23, no. 3, p. 034019, 2025.

[4] J. Endres *et al.*, "Valence and conduction band densities of states of metal halide perovskites: a combined experimental–theoretical study," *J. Phys. Chem. Lett.*, vol. 7, no. 14, pp. 2722–2729, 2016.

[5] E. Kraut, R. Grant, J. Waldrop, and S. Kowalczyk, "Precise determination of the valence-band edge in x-ray photoemission spectra: application to measurement of semiconductor interface potentials," *Phys. Rev. Lett.*, vol. 44, no. 24, p. 1620, 1980.

[6] D. R. Baer *et al.*, "XPS guide: Charge neutralization and binding energy referencing for insulating samples," *J. Vac. Sci. Technol. A*, vol. 38, no. 3, 2020.

[7] M. Poupon, N. Barrier, S. Petit, and S. Boudin, "A new β-CdTeO 3 polymorph with a structure related to α-CdTeO 3," *Dalton Trans.*, vol. 46, no. 6, pp. 1927–1935, 2017.

[8] F. Caballero-Briones *et al.*, "Structural analysis of Cd–Te–O films prepared by RF reactive sputtering," *J. Non-Cryst. Solids*, vol. 354, no. 31, pp. 3756–3761, 2008.

[9] D. Zagorac, H. Müller, S. Ruehl, J. Zagorac, and S. Rehme, "Recent developments in the Inorganic Crystal Structure Database: theoretical crystal structure data and related features," *Appl. Crystallogr.*, vol. 52, no. 5, pp. 918–925, 2019.
